# In vivo antitumor activity of doxorubicin loaded on chitosan functionalized Pb_2_Mn_2_Fe_12_O_22_ magnetic nanoparticles

**DOI:** 10.1038/s41598-025-20184-6

**Published:** 2025-10-02

**Authors:** Aliaa M. Radwan, Afaf El-Atrash, Maha Elkholy, Nagat Fawzy Nawar, Ehab Tousson, Amina I. Ghoneim

**Affiliations:** 1 Biochemistry Division, Chemistry Department, Faculty of Science, Tanta University, Tanta, 31527 Egypt; 2 Zoology Department, Faculty of Science, Tanta University, Tanta, 31527 Egypt; 3 Central Lab, Faculty of Science, Damanhour University, Damanhour, Egypt; 4 Physics Department, Faculty of Science, Tanta University, Tanta, 31527 Egypt

**Keywords:** Ehrlich solid tumor, Doxorubicin, Chitosan-coated ferrite nanoparticle, Apoptosis, Antioxidant, Drug delivery, Biochemistry

## Abstract

Nanoscale materials can improve cancer treatment by safely and efficiently delivering chemotherapeutic drugs. This study was designated to load the anticancer drug doxorubicin (DOX) into chitosan-coated Pb2Mn2Fe12O22 magnetic nanoparticles (CT-MNPs) and compare their physicochemical and biological effects with free drug, in addition to the therapeutic role of DOX-CT-MNPs to acting efficaciously in restraint of cancer cells growth and evolution using Ehrlich solid tumor model (EST). Forty female mice were randomly and equally split into four groups (EST; EST + Free DOX; EST + CT-MNPs; and EST + DOX-CT-MNPs). Our findings show that treating EST with DOX, either free or loaded on CT-MNPs, inhibits tumour growth by producing oxidative stress, disrupting the antioxidant system, activating apoptosis, and arresting the cell cycle. Furthermore, DOX loaded on CT-MNPs had greater anticancer activity than DOX in its free form. This highlights the potential advantages of CT-MNPs in tumour therapy and drug delivery.

## Introduction

Cancer, a non-communicable disease, poses a significant public health threat to both developed and developing countries^1,2^. Globally, breast cancer is the most frequent cancer type and the second foremost reason of cancer-related death among women^3^. Ehrlich solid tumor (EST) is a mouse breast cancer model that is commonly used for cancer research due to its high resemblance to human tumors and high sensitivity to chemotherapeutic drugs^4–6^. Traditional cancer therapies like radiation, surgery, and chemotherapy have improved patients’ survival. However, their effectiveness in treating advanced metastatic tumors is limited^7^.

Although doxorubicin (DOX) is the first clinically licensed medication for solid tumors, transplantable leukemias, and lymphomas, its non-specific toxicity to healthy tissues limits its clinical utility^8,9^. As a result, it is now crucial to optimize drug delivery methods to target the intended cancer site while minimizing adverse effects and improper dosages^10^. Effective targeted therapeutics can be developed on an agile platform using nanotechnology to achieve desired results. Nanoparticles (NPs) are important materials for various biomedical applications on account of their small size and inherent properties^11,12^. Magnetic nanoparticles (MNPs), multipurpose platforms that have garnered significant attention for biological applications and engineering like delivery systems for drugs and biosensors, are among the numerous inorganic NPs investigated^13^. Even though MNPs are biocompatible, chemically stable, and have a high saturation magnetization, there are obstacles to their widespread use as drug delivery systems. MNPs’ aggregation and short-term stability in biological suspension are caused by their remarkably high surface-to-volume ratio and van der Waals forces. Additionally, their circulation time is shortened after being cleared by the macrophage system^14^. These concerns are frequently addressed by functionalizing the NPs with biodegradable and biocompatible polymers, like chitosan, to extend their retention period in circulation and, consequently, their delivery effectiveness^15^.

Chitosan is one of the most widely studied and naturally existing polymers that have garnered a lot of interest in the pharmaceutical and biomedical applications for its low immunogenicity and biodegradability^16^. Furthermore, it has a diversity of reactive functional groups such as amino and hydroxyl groups that can facilitate the conjugation of imaging agents, medicines, and targeted ligands^17^.

Thus, this study was designed to load the anticancer drug doxorubicin (DOX) into chitosan-coated ferrite nanoparticles (CT-MNPs) and compare their physicochemical and biological effects with free drug. To further investigate the therapeutic and anti-tumor potential of newly synthesized DOX-CT-MNPs Nano-system against EST growth and development.

## Materials and methods

### Materials

Doxorubicin HCl (50 mg) was obtained from Hikma Pharmaceuticals (Badr city, Cairo, Egypt). Chitosan was obtained from Alpha Chemica, India.

### Preparation of Y-Type Pb_2_Mn_2_Fe_12_O_22_ Hexagonal Ferrite nanoparticles (MNPs)

The nanocrystalline Pb_2_Mn_2_Fe_12_O_22_ Y-Type Hexagonal ferrite nanoparticles (MNPs) were fabricated by the chemical co-precipitation approach^18^.

### Functionalization of Pb_2_Mn_2_Fe_12_O_22_ MNPs with chitosan (CT)

CT functionalized MNPs were made with changes from the ones published before^13^. A 0.5% chitosan (CT) solution (pH 5) was mixed with 0.5 g of Y-Type hexagonal ferrite MNPs. The mixture was then sonicated at 60 °C for 1 h and then stirred for 18 h at room temperature. The black homogenous mixture (CT-MNPs) obtained was split by an external magnetic field and dried overnight at room temperature.

### Encapsulation of doxorubicin (DOX)

DOX encapsulation was taken from that formerly published, with modifications^19^. Approximately 0.5 g of CT-MNPs were added to 50 mL of Phosphate Buffered Saline (PBS), pH 7.4. The mixtures were gently stirred at 37 ºC for 3 h, followed by the addition of 100 mg of DOX to each nanoparticle suspension. The resulting mixtures were then mechanically stirred for 8 h at an ambient temperature, then sonicated for 3 h. The DOX encapsulated CT-MNPs were separated from the suspension using an external magnet. The samples were then further washed with distilled water to remove the residual unbound DOX and dried overnight at room temperature. The sample was designated as DOX-CT-MNPs.

The amount of DOX in the DOX-loaded CT-MNPs was measured using a spectrophotometer (Shimadzu 2100S UV–Vis spectrophotometer, Japan) at 480 nm that corresponds to the absorption peak of DOX. The encapsulation efficiency was estimated using the following equation.

$${\text{Efficiency}}\,{\text{of}}\,{\text{Encapsulation }}\,\left( \% \right)\, = \frac{\begin{gathered} \left( {{\text{DOX}}.{\text{Total}}} \right) \hfill \\ - \left( {{\text{DOX}}. {\text{Free}}} \right) \hfill \\ \end{gathered} }{{\left( {{\text{DOX}}.{\text{Total}}} \right)}}\, \times \,100$$

### In vitro drug release

The in vitro release of DOX-CT-MNPs was investigated via the dialysis bag method^20^. 40 mg of DOX loaded CT-MNPs were sonicated for 1 h in 10 ml of PBS solution at pH 7.4 or pH 4.8. The dispersion was dialyzed against 200 ml PBS solution (pH 7.4 or pH 4.8) at 37 °C. The absorbance at 480 nm was measured in 2 ml of release medium at different time intervals. The quantity of released DOX was quantified using a DOX calibration curve in PBS.

### Characterization of nanoparticles

The surface morphology of nanoparticles was conducted using a JeoL-JSM- 6510 scanning electron microscope (SEM). The crystalline nature of the prepared nanoparticles was confirmed by X-ray diffraction analysis (XRD) using GNR APD 2000 Pro X-ray diffractometer step scan type and CuK_α1_ radiation with wavelength λ = 1.540598 Å. In addition, FTIR spectra were monitored at room temperature using a Bruker Tensor 27 FT-IR spectrometer in the range of 200–4200 cm^−1^ to verify the DOX-CT-MNPs synthesis. Moreover, Hysteresis loops of the samples were monitored at room temperature by the vibrating sample magnetometer, Lakeshore–7410 and a maximum applied field up to 20,000 Gauss.

### Experimental animals

The study involved 40 Swiss albino mice from the Egypt Vaccine Company’s animal house colony in Egypt. The mice were housed under ambient conditions, with a commercial diet and water supply. All experimental protocols were approved by the institutional research ethics committee of Tanta University, Faculty of Science (IACUC-SCI-TU-0223)^21^. All methods were carried out in accordance with NIH guidelines and regulations. All methods are reported in accordance with ARRIVE guidelines^22^.

### Induction of Ehrlich solid tumour (EST)

The Egyptian National Cancer Institute (Cairo University, Egypt) supplied the mice that carried Ehrlich ascites carcinoma (EAC). To sustain the EST, viable EAC cells (2.5–3 × 10^6^ cells/mouse) were embedded subcutaneously into the left thigh of each mouse according to a previously reported method^23^.

### Experimental design

After two weeks of tumor cell inoculation, forty mice (20-25g) were randomly allocated into equal four groups. The animals underwent the following treatment protocols:

- EST group: (Positive control group): mice carrying EST and received saline.
- EST + Free DOX group: mice carrying EST and treated with DOX in a dose of 4 mg/kg^24^.
- EST + CT-MNPs group: mice carrying EST and treated with suspension of magnetic ferrite nano particles coated by Chitosan (CT-MNPs) in a dose of 24 mg/kg
- EST + DOX-CT-MNPs group: mice carrying EST and treated with suspension of DOX loaded on CT-MNPs in a dose of 24mg/kg

Overall, mice were given the drugs orally every 3 days over two weeks.

### Tumor samples preparation

Following completion of the prior treatments, mice were anesthetized with isoflurane (1–4%) and sacrificed via cervical dislocation. The tumor tissues were then dissected and weighed. Tumor samples were fixed for 16 h in paraformaldehyde solution before being put into paraffin wax. Tumor specimens were then produced from the paraffin blocks for histopathologic staining with hematoxylin and eosin (H&E) as well as immunohistochemistry staining for PCNA and caspase 3. Tumor samples were immediately frozen and kept for further biochemical, flow cytometric analysis, and qPCR as detailed subsequently.

### Evaluation of lipid peroxidation and antioxidants

Using kits procured from Biodiagnostic Company, Egypt, the level of MDA as an oxidative stress biomarker in tumor homogenate was assessed using manufacturer-recommended approaches^25^. Reduced glutathione (GSH) content was also measured in the tumor homogenates using Beutler’s method. This method involves reducing 5, 5′-dithiobis-2-nitrobenzoic acid (DTNB) by GSH to produce a yellow chromophore with an absorbance of 412 nm^26^. Additionally, catalase activity (CAT) was determined by measuring the breakdown of H_2_O_2_ at 240 nm, as stated by Beers^27^. The activity of superoxide dismutase (SOD) was evaluated in EST homogenate depending on the autoxidation of adrenaline as previously reported^28^.

### Cell cycle analysis

Flow cytometric measurements were utilized to determine cell cycle distribution through different stages. Tumor samples taken from non-treated and tumor-bearing treated groups were crushed after being snap frozen in liquid nitrogen. The cells were probed with propidium iodide and kept for 1 h in the dark. A cell cycle profile study was carried out by an Accuri C6 flow cytometer, and apoptotic cells were recognized as a sub-G1 peak. using the cell Quest analysis program (Becton Dickinson, Sunnyvale, CA, USA)^29^.

### Apoptosis determination by flowcytometry

The proportion of cells that go through apoptosis was measured using Annexin V/PI double staining kit (Immunostep, Spain) according to the manufacturer’s procedure. Tumor tissues from all experimental groups were broken down after being freeze-dried in liquid nitrogen. Following a wash with PBS, the tumor cells were suspended in an appropriate binding buffer. Annexin-V/PI staining solution was then mixed with the cell suspension and kept in the dark for 15 min. The samples were subsequently examined using flow cytometer (Becton Dickinson)^30^.

### Quantitative real-time polymerase chain reaction (qRT-PCR)

The total RNA from the tumor tissues of the untreated and treated EST groups was extracted and purified using an RNA extraction kit (Thermo Scientific, Fermentas, K0731). The quantity of RNA was estimated by a Uv–Vis spectrophotometer Q5000/USA. Reverse transcription kits (Thermo Scientific, Fermentas, EP0451) were used to synthesize complementary DNA (cDNA) from 5 µg of RNA, following manufacturer specifications. qPCR was utilized to evaluate the relative expression levels of PTEN, GSK-3β, and MAPK1 using 2X Maxima SYBR Green/ROX qPCR Master Mix (Thermo Scientific, USA, K0221). Glyceraldehyde 3-phosphate dehydrogenase (GAPDH) has been employed as an internal control. The data was evaluated using the ΔΔCt comparative technique, with the fold of change = 2^−ΔΔCt^^31^.

### Histopathological examination

Tumor specimens from various groups were removed and fixed in 10% neutral buffered formalin before being paraffin embedded and sectioned and then stained with hematoxylin and eosin. The histology of tumor tissues was examined using a light microscope^32^.

### Immuno-histochemical analysis

Immuno-labeling involved loading 5 μm slices of each tumor onto positively charged slides. Different monoclonal anti-mouse primary antibodies were used to detect caspase-3 and proliferating cell nuclear antigen (PCNA). The correct secondary antibodies were used to localize the bound primary antibodies, and the Diaminobenzidine (DAB) stain was used in accordance with the manufacturer’s protocol^33^.

### Statistical assessment

Data were expressed as the significance of difference was analysed by one–way ANOVA. Values are expressed as means ± SE. (a) and (b) significant difference from control and from EST group respectively at *P* < 0.05. The power of the sample size was calculated using post-hoc power calculator online server. The power value was 0.9 which is considered adequate and indicate the chance of detecting differences between groups.

## Results

### Characterization of the prepared DOX-chitosan-magnetic nanoparticles (DOX-CT-MNPs)

**Fig. 1:**
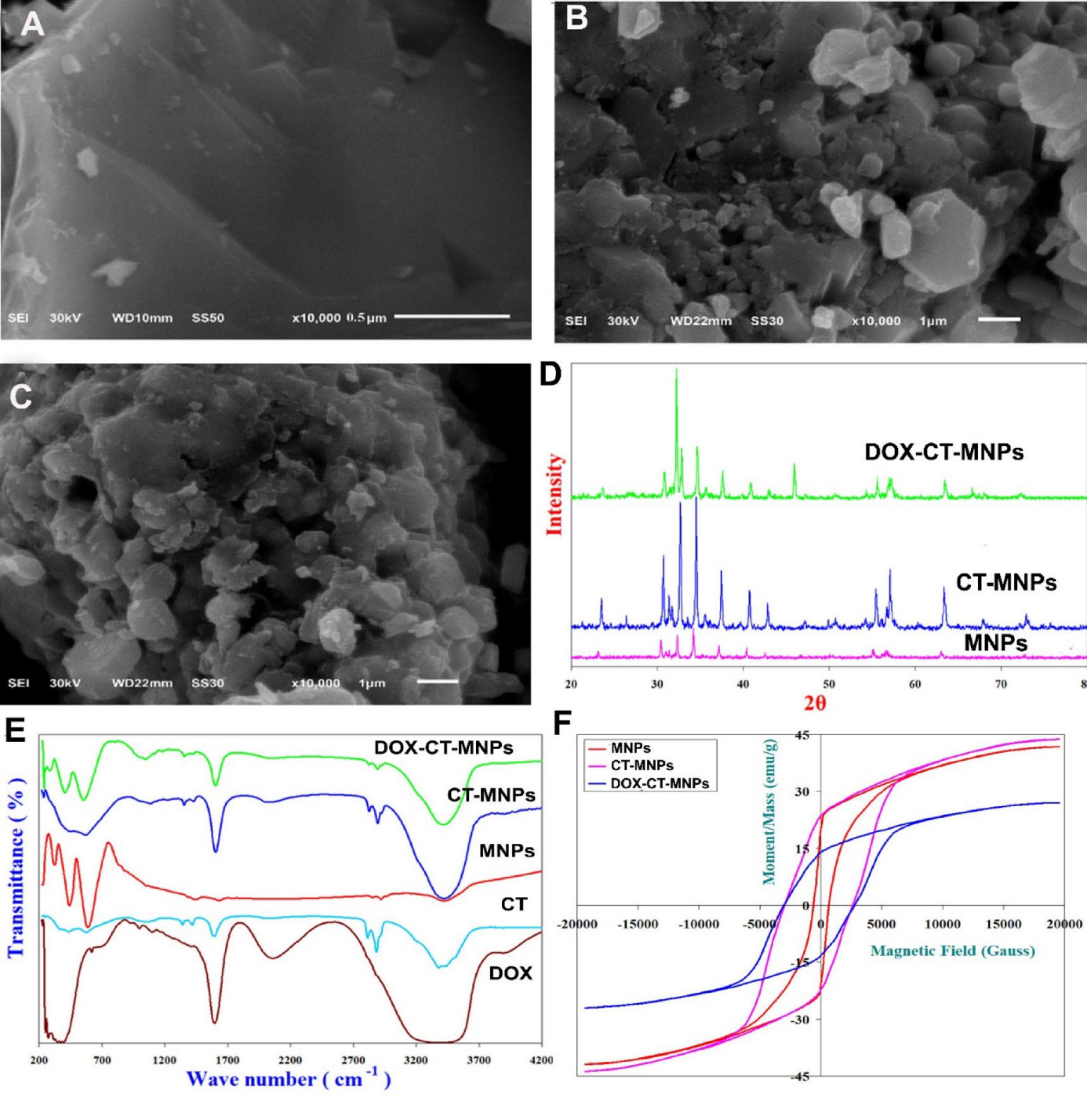
SEM images for (**A**) MNPs, (**B**) CT-MNPs, and (**C**) DOX-CT-MNPs. (**D**) XRD pattern. (**E**) FTIR absorption spectra. (**F**) Magnetization curve for the prepared nanoparticles.

The characterization results of the prepared nanoparticles were illustrated in Fig. 1. SEM micrographs of MNPs, chitosan coated MNPs (CT-MNPs), and DOX loaded MNPs (DOX-CT-MNPs) showed hexagonal morphology. CT-MNPs revealed a number of coarse-grained particles and an increase in crystal size, implying a coating effect (Fig. 1A, B and C). XRD patterns for the nanocrystalline Pb_2_Mn_2_Fe_12_O_22_ Y-Type Hexagonal ferrite nanoparticles (MNPs), CT-Pb_2_Mn_2_Fe_12_O_22_ (CT-MNPs) and DOX-CT-Pb_2_Mn_2_Fe_12_O_22_ (DOX-CT-MNPs) nano-system was illustrated in Fig. 1D. XRD patterns verified that the resultant composites have single-phase Y-Type Hexagonal nano ferrites which agrees well with JCPDS data (Card number 00-054-0069) [3–5]. XRD patterns of Pb_2_Mn_2_Fe_12_O_22_ Y-Type coated with chitosan showed some broadening in the diffraction peaks as a result to the amorphous nature of chitosan and indicating that the Pb_2_Mn_2_Fe_12_O_22_ Y-Type nanostructures is coated by the chitosan matrix. Furthermore, the chitosan as well as DOX do not induce phase change of Pb_2_Mn_2_Fe_12_O_22_ Y-Type nanostructures. Also, the packaging has increased only for 2θ = 32.27. Obviously, the use of a chitosan-based cross-linked network with the Pb_2_Mn_2_Fe_12_O_22_ Y-Type nanostructures and the formation of DOX-CT-MNPs nano-system induced a widening of the emerging peaks. The grain size was calculated from XRD data to be 37, 68 and 74 nm for MNPs, CT-MNPs, and DOX-CT-MNPs respectively.

Figure 1E illustrates IR spectra for the nanocrystalline Pb_2_Mn_2_Fe_12_O_22_ Y-Type Hexaferrite, where 8 absorption bands; ν_1_, ν_2_, ν_3_, ν_4_, ν_A_, ν_B_, ν_T1_ and ν_T2_ are detected. Three characteristic bands (ν_1_, ν_2_ and ν_3_) confirmed Y-Type Hexaferrite. ν_1_ at 592.12 cm^−1^, ν_2_ at 447.47 cm^−1^ and ν_3_ at 329.8 cm^−1^ are set to intrinsic stretching vibrations of A-site metal ion-oxygen bonding, intrinsic vibrations of B-site metal ion oxygen complexes and to divalent ion bonds Pb^2+^–O^2-^, Mn^2+^–O^2-^ and Fe^2+^–O^2-^ were amongst B-sites, respectively. ν_4_ is spotted at of 235.3 cm^−1^ and set to lattice vibrations of the system. ν_T1_ at 1647.16 cm^−1^ is referred to the existence of retained water in samples. The typical chitosan peaks around 1444.65 and 1612.45 cm^−1^ for amino and amide groups which are weak as a result for the presence of chitosan in a small ratio with respect to Pb_2_Mn_2_Fe_12_O_22_ nanoparticles [12]. Bands of Chitosan (CT) observed at 3444.77 cm^−1^ related to axial stretching of O–H group, which appears superimposed to N–H stretching band. For CT, the C = O stretching of acetyl units at 1612.45 cm^−1^. Bending vibrations for N–H group at 1444.65 cm^−1^. Specific bands of C-O and C–O–C stretching absorbing at 1105.18 cm^−1^ were noted for CT. Thence, CT have perfectly coated Pb_2_Mn_2_Fe_12_O_22_ nanoparticles. IR spectrum of DOX-CT-MNPs confirmed the main characteristics bands of DOX at 3444.78, 2902.79, 2846.86, 1614.38, 1444.65, 1058.89, 825.51 and 624.92 cm^-1^ corresponding for N–H stretch, C–H stretch, CH_2_ stretching, C = O stretch, C = C ring stretch, C–O–C stretch, C = H bend and C = C ring bend, respectively.

In addition, the magnetic characteristics of prepared nanoparticles were displayed in Fig. 1F. Room temperature hysteresis loops for the nanocrystalline Pb_2_Mn_2_Fe_12_O_22_ Y-Type Hexagonal ferrite nanoparticles indicated a wide hysteretic behavior (high coercivity Hc) which is characteristic of hard magnetic materials, with high saturation magnetization Ms = 41.854 emu/g, high coercivity Hc is 642.65 Gauss, high remanent magnetization Mr = 20.401 emu/g and high squareness Mr/Ms = 0.48745. The uncoated Pb_2_Mn_2_Fe_12_O_22_ exhibited a high saturation magnetization (MS), which enhanced with chitosan (CT) coating to 43.774 emu/g. CT-MNPs revealed a substantial shielding effect by chitosan. Hc were found to increase after coating with CT reaching 2797.7 Gauss. Materials with strong magnetic anisotropy typically have high coercivity, meaning they are difficult to demagnetize. Hc increased upon CT coating, conferring a hardening or binding effect on Pb_2_Mn_2_Fe_12_O_22_. Conversely, a reduction was noticed in all magnetic Properties (Ms = 27.045 emu/g and Hc = 2868.3 Gauss) for DOX-CT-MNPs, which may be attributed to the reduced surface defects on Pb_2_Mn_2_Fe_12_O_22_ and the dilution effect of DOX.

### Loading and in-vitro release of DOX from the prepared NPs

**Fig. 2:**
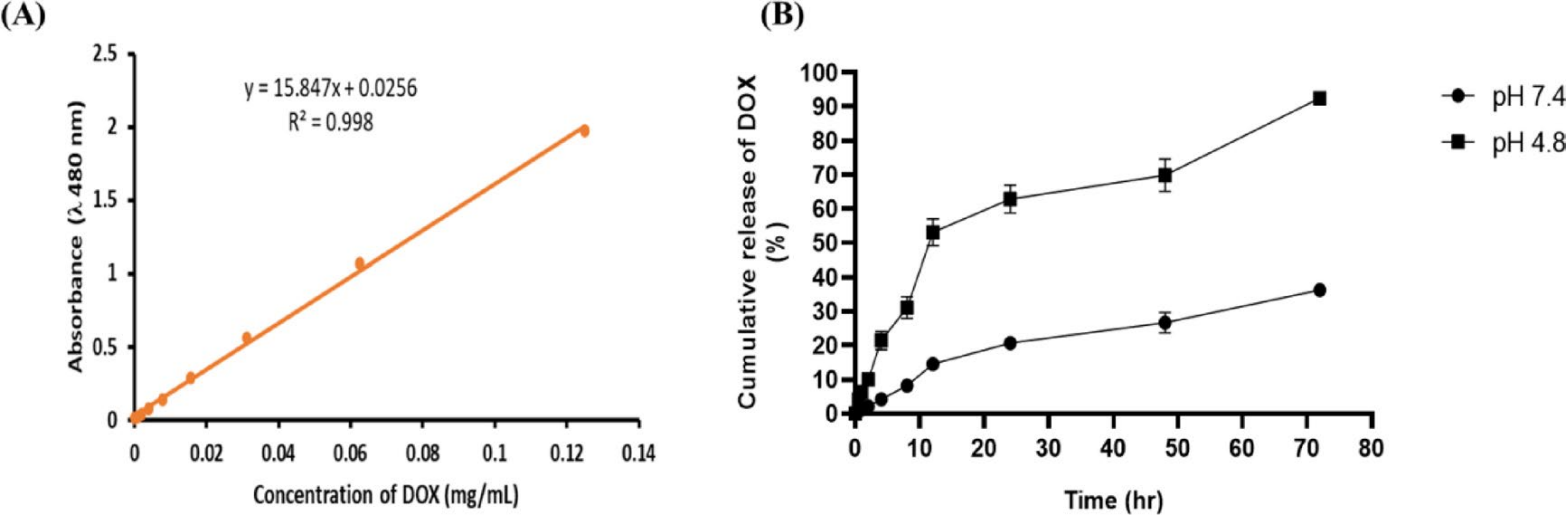
In-Vitro release profile of DOX. (**A**)Standard curve of DOX. (**B**) DOX release from DOX-CT-MNPs formula at pH 7.4 and pH 4.8. Data are displayed as Mean ± SD (n = 3).

The dialysis bag approach was utilised to investigate the DOX release from DOX-CT MNPs. Figure 2A depicts the calibration curve of doxorubicin at a known concentration in physiological conditions (PBS, pH 7.4). Figure 2B illustrates the release profile of DOX; within 72 h, about 92.54% of DOX was released at pH 4.8 (cancer pH). In contrast, the rate of DOX release at pH 7.4 (normal pH) was roughly 36.27% over 3 days, which is significantly slower than that at the cancer pH. Furthermore, the drug loading content in the DOX-CT-MNPs was reliably determined to be 83.1%

### Changes in tumor weight

**Fig. 3:**
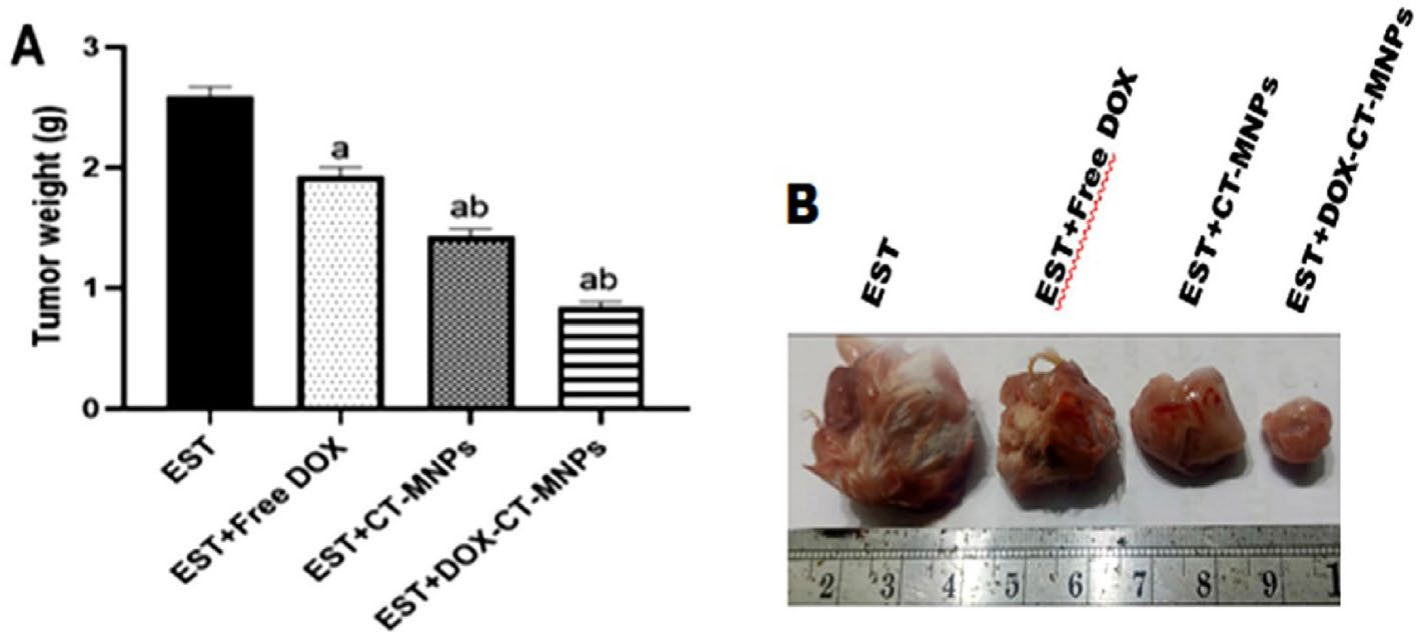
Influence of free DOX, CT-MNPs, and DOX-CT-MNPs on the development of tumor weight and size. (**A**): Histogram of tumor weight in different experimental groups. Data represented as mean ± SD (n = 10). One-way ANOVA followed by post-hoc Tukey’s test was done. *P* < 0.05 is statistically thought a significant, where ^a^ significant difference from EST group and ^b^ significant difference from EST + free DOX group. (**B**): Images of tumor tissues excised from different experimental groups.

Figure 3 revealed that treating solid tumors implanted in mice with free DOX, CT-MNPs, and/or DOX-CT-MNPs resulted in significant reductions (*P* < 0.01) in tumor weight and size compared to the EST group. The DOX-CT-MNPs treatment group showed the highest decrease ratio compared to the free DOX or CT-MNPs groups.

### DOX-CT-MNPs modulated EST induced alteration in lipid peroxidation and antioxidant markers

Oxidative stress is among the cytotoxic machinery triggered by doxorubicin. In this investigation, the levels of thiobarbituric acid reactive substances (TBARS) as oxidative exertion indicator, glutathione (GSH) content, superoxide dismutase (SOD), and catalase (CAT) antioxidant enzymes were determined in various experimental groups treated with either DOX in free form or loaded on CT-MNPs. The data exhibited in Table 1 revealed that EST mice that received free DOX, CT-MNPs, or DOX-CT-MNPs had a substantial (*P*˂0.0001) increase in TBARS associated with a notable (*P*˂0.0001) drop in GSH, SOD, and CAT compared to the untreated EST group. Moreover, DOX-CT-MNPs treated group exposed a noteworthy elevation in TBARS oxidative level, GSH, and SOD compared to DOX treated group.

**Table 1:** Changes in oxidative stress and antioxidant parameters in Ehrlich solid tumor bearing mice treated with free DOX and DOX-CT-MNPs.

Groups	TBARS (nmol/g tissue)	GSH (mmol/g tissue)	SOD (U/g tissue)	CAT (U/g tissue)
EST	8.94 ± 0.15	0.62 ± 0.003	53.35 ± 0.4	23.02 ± 0.37
EST + Free DOX	14.08^a^ ± 0.36	0.32^a^ ± 0.012	39.06^a^ ± 0.6	11.32^a^ ± 0.04
EST + CT-MNPs	12.51^a^ ± 0.26	0.47^a^ ± 0.018	42.56^a^ ± 0.6	14.95^a^ ± 0.38
EST + DOX-CT-MNPs	18.13^ab^ ± 0.11	0.26^ab^ ± 0.004	34.19^ab^ ± 0.4	10.29^a^ ± 0.78

Data were presented as mean ± SD (n = 10). One-way ANOVA followed by post-hoc Tukey’s test was done. *P* < 0.05 is statistically considered significant, where ^a^ significant difference from EST group and ^b^ significant difference from EST + Free DOX group.

### DOX-CT-MNPs impact the cell cycle in Ehrlich solid tumor bearing mice

**Fig. 4:**
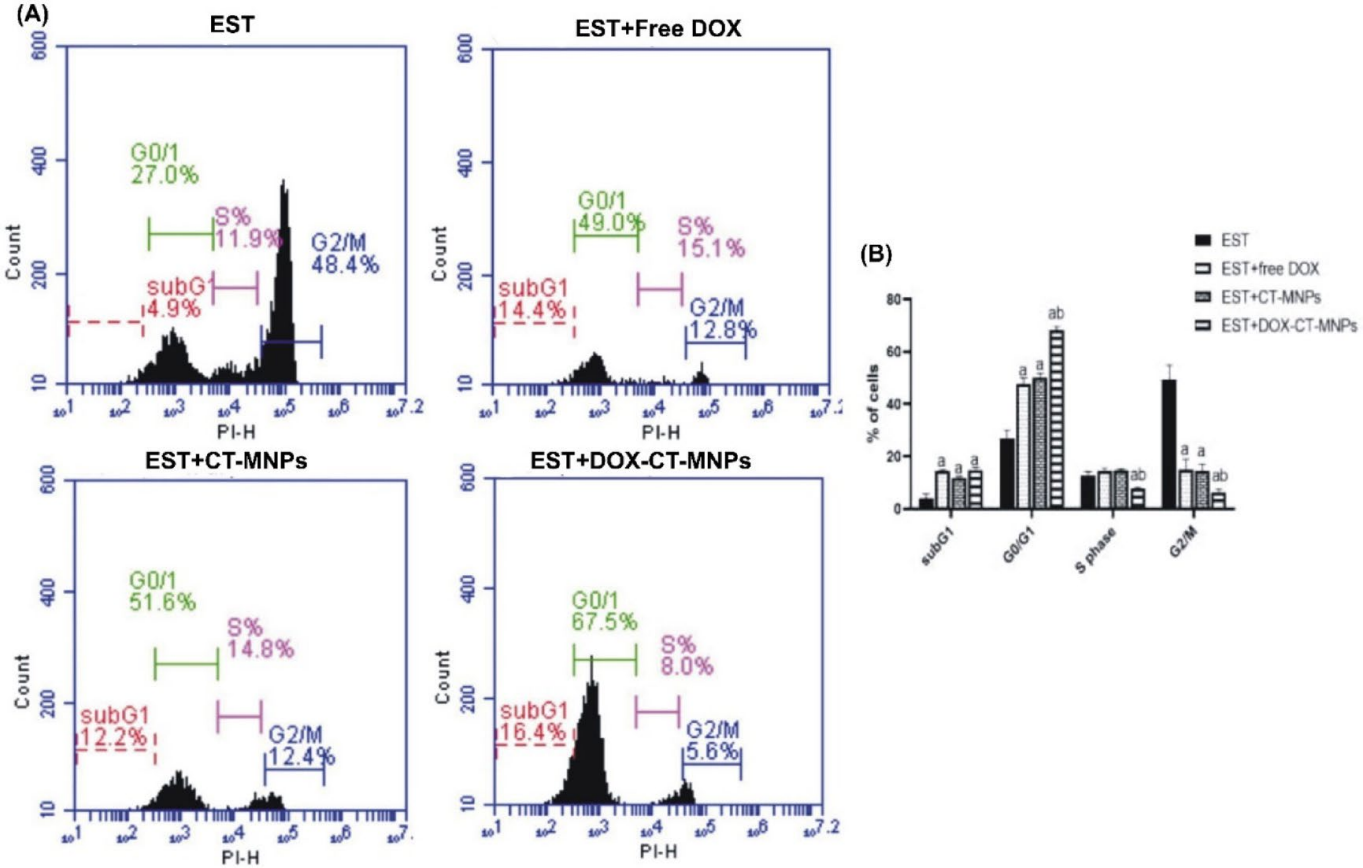
Flow cytometric investigation of cell cycle arrest by free DOX and DOX-CT-MNPs in Ehrlich solid tumor mice model. (**A**) Cell distribution at different phases with and without treatment. (**B**) Graph depicting the distribution of cells in different phases. Data represented as mean ± SD (n = 10). Two-way ANOVA followed by post-hoc Tukey’s test was done. *P* < 0.05 is statistically thought a significant, where ^a^ significant difference from EST group and ^b^ significant difference from EST + free DOX group.

The cell cycle was investigated by measuring the content of the DNA in tumor cells labelled with PI flow cytometrically (Fig. 4). Based on the findings, in comparison to EST group, free DOX, CT-MNPs, and DOX-CT-MNPs promoted G0/G1 phase arrest, evidenced by an increase in the G0/G1 phase cell population to 49%, 51.6%, and 67.5% respectively. In addition, free DOX, CT-MNPs, and DOX-CT-MNPs had 14.4%, 12.2%, and 16.4% of the population in subG1 respectively, compared to 4.9% in EST group. Further, the treatment of EST with free DOX and DOX-CT-MNPs significantly (*P˂0.0001)* diminished the G2/M phase population to 12.8% and 5.6% respectively, compared to 48.4% in EST untreated group.

### DOX-CT-MNPs induce apoptosis in Ehrlich solid tumor bearing mice

**Fig. 5:**
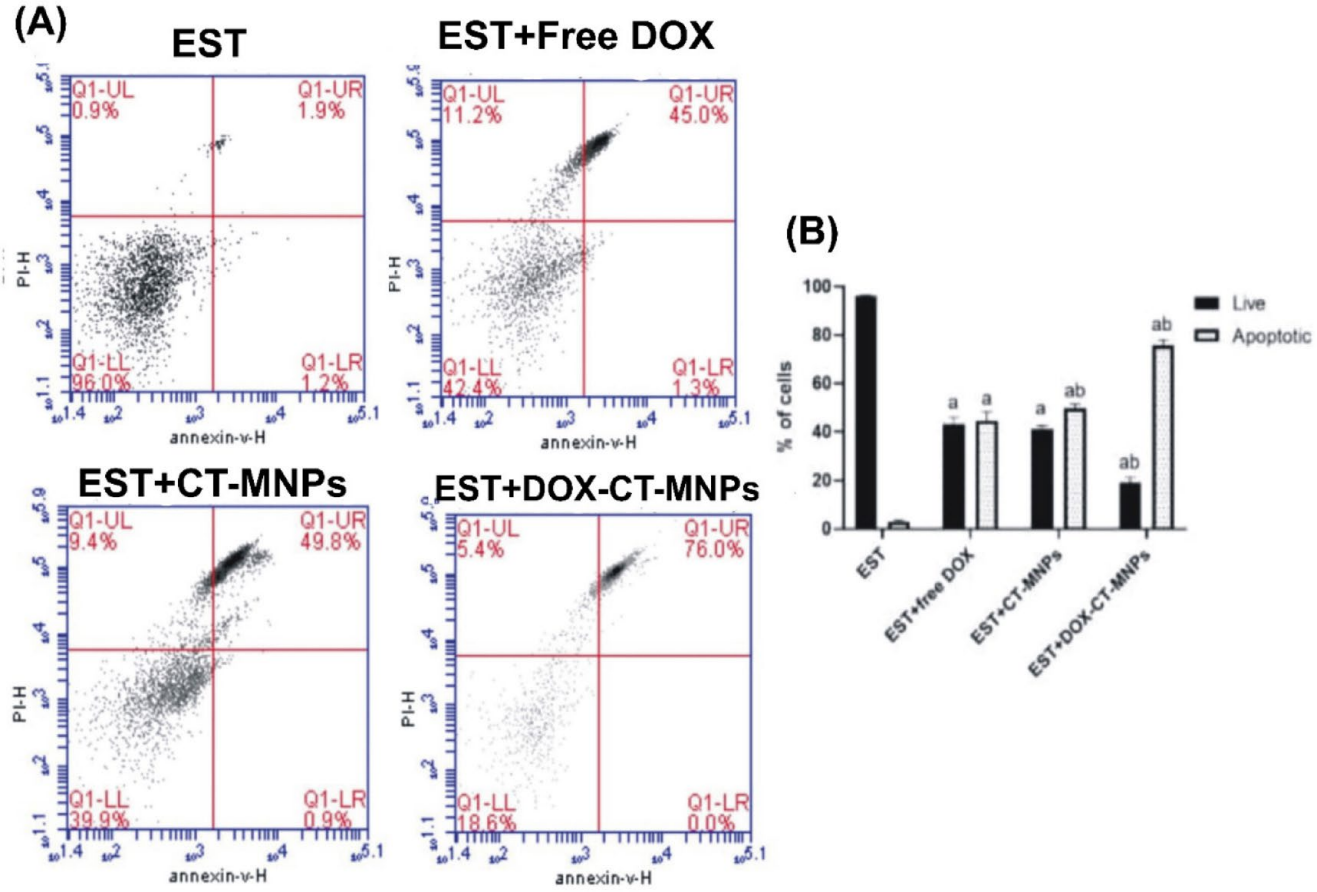
Flow cytometric assessment of apoptosis mediated by free DOX and DOX-CT-MNPs in EST-bearing mice. (**A**) Representative histogram of Annexin V and PI staining showing the population of viable and apoptotic cells. (**B**) Graph depicting the percentage of viable and apoptotic cells in EST treated with free DOX or DOX-CT-MNPs. Data represented as mean ± SD (n = 10). Two-way ANOVA followed by post-hoc Tukey’s test was done.* P* < 0.05 is statistically counted a significant, where ^a^ significant difference from EST group and ^b^ significant difference from EST + free DOX group.

Apoptosis is a vastly controlled method of cell death characterised by a multitude of metabolic changes and different cellular shape. In this study, the effects of DOX-CT-MNPs cell death in tumours collected from the DOX-CT-MNPs and free DOX-treated groups were compared to those of the Ehrlich solid tumour (EST) untreated group using flow cytometry with Annexin V-FITC/PI (Fig. 5). In Ehrlich solid tumor treated with free DOX, CT-MNPs, or DOX-CT-MNPs, the percentage of viable cells decreased dramatically (*p* < 0.0001) compared to the EST untreated group. Otherwise, DOX, CT-MNPs, and DOX-CT-MNPs treatments significantly (*p* < 0.0001) enhanced the proportion of apoptotic cells compared to the untreated group (EST). The effect of cell death induced by DOX-CT-MNPs was more prevalent than that of free DOX treatment, as the percentage of apoptotic cells was 75.75% in EST + DOX-CT-MNPs compared to 44.5% in EST + DOX treated group.

### DOX-CT-MNPs affect PTEN, GSK-3β, and MAPK1 expression in Ehrlich solid tumor bearing mice

**Fig. 6:**
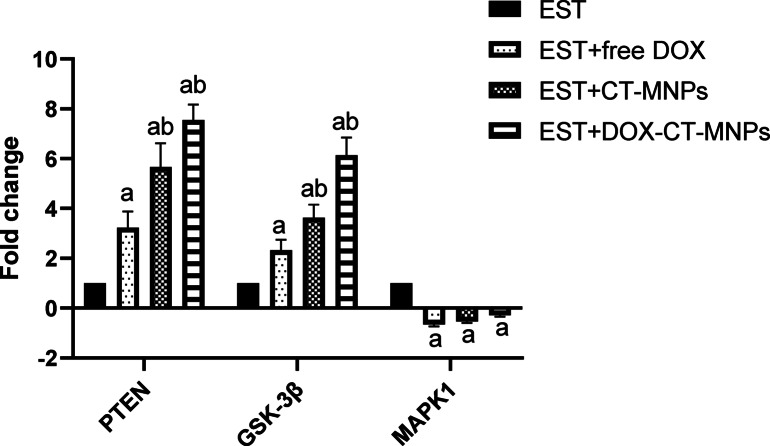
Changes in expression levels of *PTEN, GSK-3β*, and *MAPK1* in EST- bearing mice treated with free DOX and DOX-CT-MNPs. The mRNA level was detected using qPCR. Data represented as mean ± SD (n = 10). Two-way ANOVA followed by post-hoc Tukey’s test was done. *P* < 0.05 is statistically recorded as a significant, where ^a^ significant difference from EST group and ^b^ significant difference from EST + free DOX group.

Figure 6 shows that doxorubicin treatment enhanced mRNA expression levels of *PTEN* and *GSK-3β* in Ehrlich solid tumor. In contrast, a remarkable (*P*˂0.01) reduction in *MAPK1* expression level was noticed in EST group treated with free DOX. In addition, DOX-CT-MNPs treated group demonstrated a significant (*P*˂0.0001) elevation in *PTEN* and *GSK-3β* expression compared to that treated with free DOX.

### Histopathological observation and immunohistochemical detection of caspase-3 and PCNA expression

**Fig. 7:**
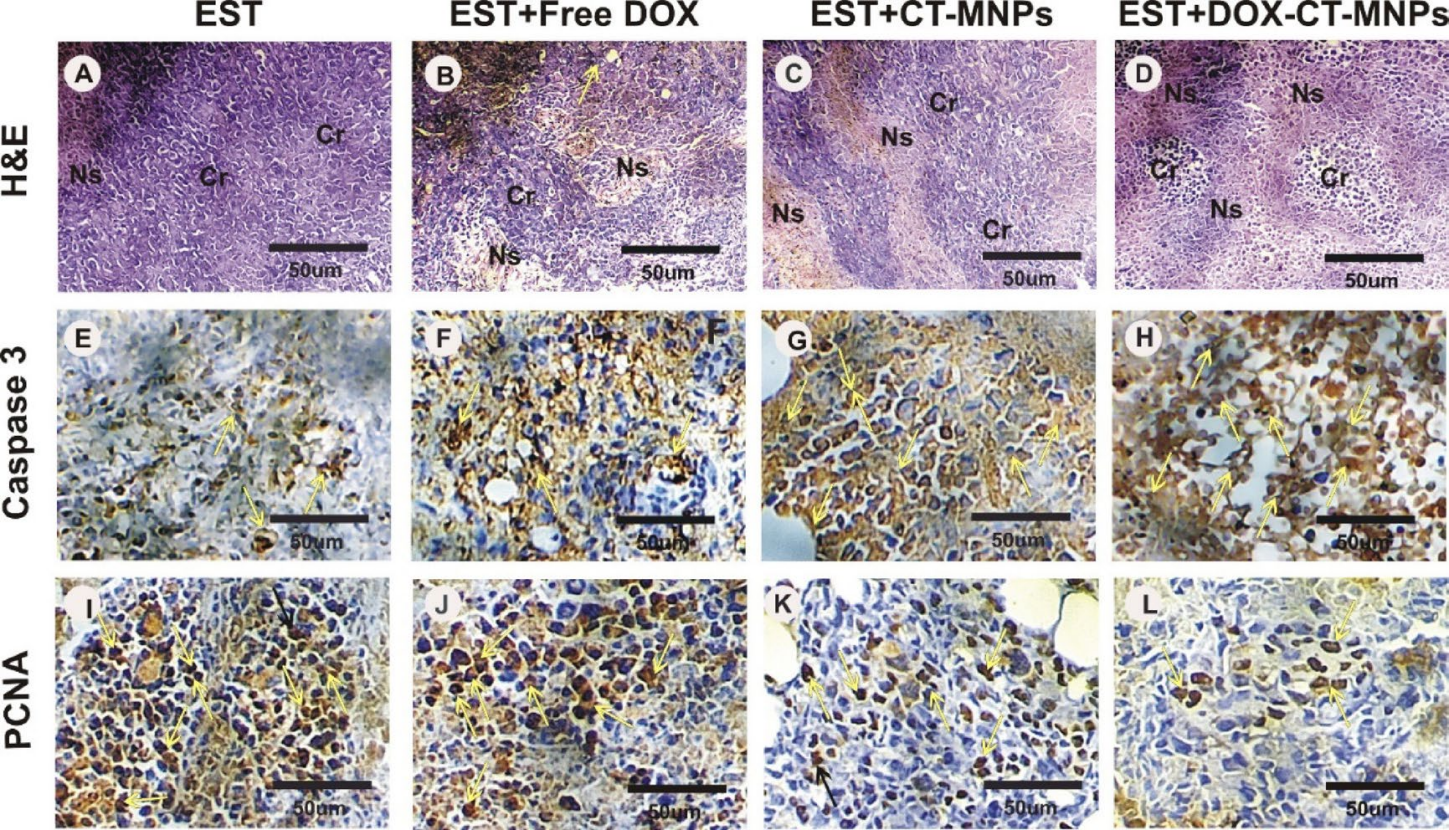
Histopathological alterations and immunohistochemical staining of caspase-3 and PCNA in Ehrlich solid tumor bearing mice treated with free DOX and DOX-CT-MNPs. (**A**- **D**) Tumor sections photomicrographs stained with HE. (**A**): EST group showing intact cancer cells (Cr) occupying 85% of the skeletal muscle bundles with only 15% necrosis (Ns). (**B**): EST + Free DOX group showing about 55% intact cancer cells and about 45% necrosis. (**C**): EST + CT-MNPs group showing about 70% intact cancer cells and about 30% necrosis. (**D**): EST + DOX-CT-MNPs group showing about 40% intact cancer cells and about 60% necrosis. (**E**–**H**) Immuno-histochemical localization of Caspase 3 protein (yellow arrows). (**I**-**L**) Immuno-histochemical localization of PCNA (Black arrows).

Histological inspection of tumor tissues in the EST group revealed the presence of intact cancer cells occupying 85% of the skeletal muscle bundles that are made up of sheets and nodules of distinctly pleomorphic viable tumor cells with hyperchromatic nuclei penetrating between devastated muscle fibers, a few scattered apoptotic cells, and small areas of necrosis about 15% (Fig. 7A). The intact cancer cells depleted with increase of necrosis areas after EST post treatment with Free DOX, CT-MNPs and/or DOX-CT-MNPs groups with best modulation and improvement in EST + DOX-CT-MNPs (Fig. 7B-D). Furthermore, the immuno-histochemical localization of Caspase 3 and PCNA in tumor tissues of different experimental groups were displayed in Fig. 7. The tumor section in EST mice displays a mild reaction for caspase 3, although mild to moderate reaction was observed in EST + Free DOX group (Fig. 7E, F). Moderate reaction for Caspase-3 was detected in EST + CT-MNPs while strong reaction was observed in EST + DOX-CT-MNPs group (Fig. 7G, H). In addition, the tumor section in EST mice indicates strong reaction for PCNA expression, although moderate reaction was observed in EST + Free DOX group (Fig. 7I, J). Moderate to mild reaction for PCNA was detected in EST + CT-MNPs while mild reaction was observed in EST + DOX-CT-MNPs group (Fig. 7K, L).

## Discussion

Cancer ranks as one of the utmost lethal diseases and leading causes of mortality worldwide, marked by unplanned and uncontrolled cellular growth^34^. Cancer therapy remains a significant difficulty, even in the twenty-first century. Current therapeutic choices, such as surgery, chemotherapy, and radiotherapy are ineffective in reducing mortality rates. Additionally, the side effects of these treatments have a negative impact on patient survival^35^. Regressive research has been ongoing for the past two decades to discover efficient cancer treatments. The iron-based magnetic nanoparticles (MNPs) are used extensively for cancer treatment and diagnosis due to their unique physical and chemical features, as well as their ability to function at both the molecular and cellular levels^36^. The aspect of the present research work is to encapsulate the antineoplastic medication DOX in chitosan functionalized ferrite MNPs and assess its antitumor potency against Ehrlich solid tumor. The initial characterization indicated that the MNPs used in this research were efficaciously created via the chemical co-precipitation process, functionalized with chitosan, and complexed with DOX. The MNPs were small and hexagonal in shape with super-paramagnetic features. The conjugation of DOX to MNPs was confirmed by SEM and FTIR. The average particle size of the resultant NPs was determined using Debye Sherrer’s equation to be less than 100 nm, indicating moderate stability and the requisite physicochemical qualities for drug delivery.

The DOX encapsulation efficiency of the produced MNPs was 83.1%, which is consistent with Ramnandan et al.’s result that CHIMgFe2O4has a DOX loading of 84.28%. The process of DOX loading into CT-MNPs involves hydrophobic and van der Waals interactions, showing that CT is a suitable biopolymer for imbuing the NPs with drug delivery capabilities ^37^. Furthermore, therelease profile results revealed that a little amount of DOX was liberated at physiological pH, a condition prevalent in healthy cells. This could considerably reduce the harmfulness in healthy cells, resulting in fewer ancillary adverse effects. Our data supportprevious findings which reported that the release of DOX from the polymeric nanocapsules was faster (~ 97% within 72 h) at pH 4.8 than at pH 7.4 (~ 97% over 23 days). This indicates the selectivity of Dox-PCL nanocapsules to be released at the cancer site (pH4.8), while Dox was hardly released at the normal site (pH 7.4)^38^

The cytotoxicity of nano-delivery carriers is vital for the antitumor medication DOX, which aims at destroying cancer cells. The surface characteristics of MNPs, whether uncoated or functionalized, play a crucial part in cytotoxicity. Direct interaction between MNPs and cells can lead to iron overload and cell death as previously reported^39^. The systemic safety of the prepared nano system against liver tissue was assessed. Our published results demonstrated that the encapsulation of DOX into CT-MNPs reduced liver injury caused by free DOX by lowering oxidative stress, apoptosis, and enhancing antioxidant parameters^21^**.** In the present work, the Ehrlich solid tumor model was utilized to test the biotherapeutic effects of DOX-CT- MNPs over free DOX. The resulting data demonstrated a substantial reduction in tumor weight and volume after treatment with DOX-CT-MNPs compared to DOX as free form. This is consistent with Ramnandan et al.’s findings that DOX-loaded MNPs exhibit greater anticancer activity than a free DOX. One of the postulated mechanisms underlying DOX’s cancer-killing actions is the inducement of oxidative stress and reactive oxygen species generation, leading to an imbalance between free radicals and the antioxidant defense system^40^. In fact, DOX was reported to bind with iron and lead to an imbalance in ferric/ferrous cycle resulted in ROS generation and antioxidant imbalance^41^. As a result, in the current study, the quantity of MDA, a byproduct of lipid peroxidation as well as reduced glutathione level, catalase and superoxide dismutase activities were assessed in tumor tissue. The results revealed that EST mice treated with free DOX or DOX-CT-MNPs had a substantial rise in MDA and a considerable depletion in GSH, CAT, and SOD compared to the untreated EST group; however the effect of DOX loaded on CT-MNPs was more pronounced than free DOX. Our findings are comparable with those of Hernandes et al., who found that DNA damage, lipid peroxidation, and ROS creation were more prominent in MCF-7 cells treated with magnetic nanoparticles containing DOX than with DOX alone^42^. As a consequence, we could conclude that free DOX or DOX loaded on ferrite nanoparticles disrupts redox balance within cancerous tissues that could induce apoptosis and cell cycle arrest.

Apoptosis is a key mechanism for maintaining homeostasis in the context of cell division and death. The improper regulation of apoptosis is known to cause the formation and propagation of cancer^43^. As a result, promoting the apoptosis in cancerous cells is a critical endeavor in the expansion of anticancer medications. DOX is an efficient chemotherapeutic medication that stimulates apoptosis in cancer cells. Flow cytometry was used to assess whether CT-MNPs may enhance DOX’s ability to trigger apoptosis in Ehrlich solid tumor in mice. In comparison to free DOX and CT-MNPs, the obtained results showed that the DOX-CT-MNPs formula significantly raised the degree of apoptosis in EST-bearing mice. Our findings were supported by a previous study that revealed DOX loaded on chitosan-superparamagnetic iron oxide (DOX-CT-SPIO) nanoparticles prompted ovarian cancer cells, A2780 and OVCAR-3, to undergo apoptosis by 91% and 87.1%, respectively^44^. Further, the apoptotic impact of the fabricated formula was confirmed by histopathology that disclosed a clear increase in apoptotic cells after being treated with DOX-CT-MNPs compared to free DOX alone. Additionally, caspase-3 was elevated with a notable decline of PCNA in EST-bearing mice treated with DOX-CT-MNPs. Based on the collected findings, we concluded that the DOX-CT-MNPs could induce apoptosis. The resultant findings are consistent with the previous report that indicated gliomas sections showed high apoptotic cells with elevation in caspase-3 after receiving DOX- superparamagnetic iron nanoparticles^45^.

Additionally, to investigate whether DOX-CT-MNPs formula able to induce cell cycle arrest beside its potential to trigger the apoptosis, cancerous cells from tumor tissues were stained by PI and examine flow cytometrically. The collected histograms indicated that either free DOX or DOX-CT-MNPS arrested the cells at G0/G1. Free Dox readily enters the nucleus and diffuse passively to the intracellular matrix. In case of DOX-CT-MNPs, DOX is liberated from the formula in lysosomes because of the acidic pH. Then, the DOX enter the nucleus and bind to the DNA causing cell cycle arrest^46^. The potential of DOX-CT-MNPs to stop the cell cycle was confirmed by Hernandes et. al who’s results suggested that DOX loaded on iron oxide nanostructure induced MCF-7 cycle blockage at G2 phase^42^.

**Fig. 8:**
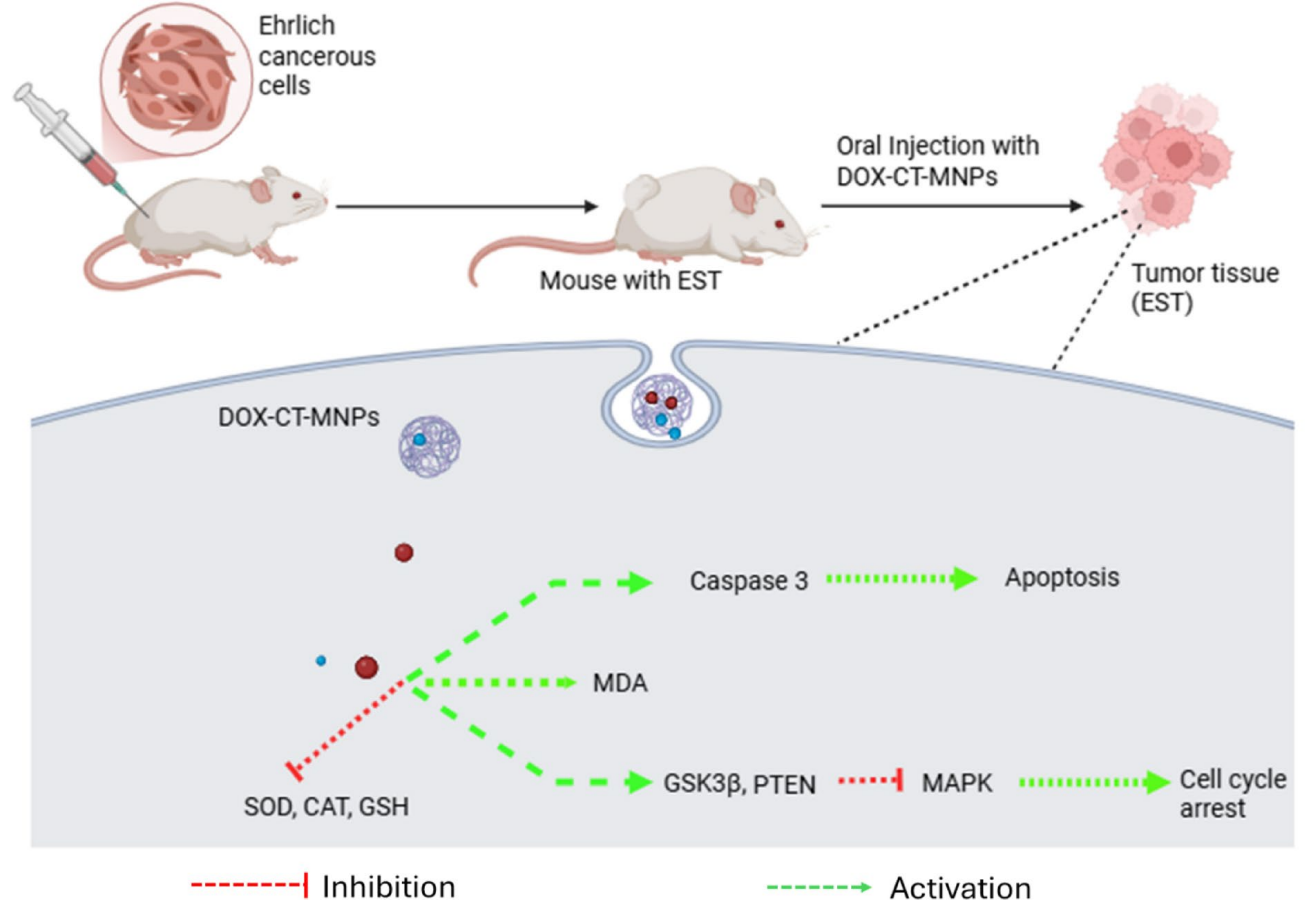
Mechanistic diagram showing the molecular changes caused by DOX-CT-MNPs. The oral administration of DOX-CT-MNPs in Ehrlich solid tumor (EST) bearing mice increase the expression of PTEN and GSK3β tumor suppressor genes that block MAPK pathway, inhibiting cell growth, proliferation, and cell cycle propagation. Also, treating EST mice with DOX-CT-MNPs increases caspase 3 expression, which causes apoptosis and cell death. Furthermore, DOX-CT-MNPs induce oxidative stress in tumour tissue by raising MDA levels while lowering GSH, CAT, and SOD activity.

PTEN (phosphatase and tensin homolog) and the MAPK (Mitogen-Activated Protein Kinase) pathways are both important in controlling cell growth and survival, and their interactions have a crucial role in cancer progression. *PTEN* acts as a tumor suppressor and also negatively regulates the *MAPK* pathway, a critical signaling cascade implicated in cell proliferation and survival. In cancer, loss of *PTEN* function can activate the *MAPK* pathway, resulting in uncontrolled cell proliferation and tumor formation^47^. Hence, the inhibition of *MAPK* pathway could be one of possible therapeutic routes of cancer. Impressively, our results indicated that DOX-CT-MNPs could inhibit growth, proliferation and cell cycle propagation via inhibiting of *MAPK1*^48,49^. Further, the current study exhibited that free DOX or DOX-CT-MNPs significantly upregulate the expression of *PTEN and GSK3β* tumor suppressor genes which further led to a decline in MAKP1 that promotes cell growth and proliferation blockage (Fig. 8)*.* Yang et al., reported that DOX inhibits Ras-ERK path in MCF-7 cancerous cells which agreed with our findings^50^.

## Conclusions

The study found that Pb_2_Mn2Fe_12_O_22_ nanoparticles (MNPs) efficiently contained doxorubicin, with chitosan acting as a stabilizing and functionalizing factor. Chitosan-coated magnetic nanoparticles contained around 83.1% of doxorubicin, indicating a considerable increase in drug loading. DOX loaded on chitosan coated magnetic nanoparticles increased the drug’s efficacy and antitumor potential against Ehrlich solid tumor-bearing mice by increasing oxidative stress, apoptosis, disrupting the antioxidant system, and arresting cell cycle. Thus, Pb2Mn2Fe12O22 magnetic nanoparticles coated with chitosan can serve as an effective medication delivery strategy. Further preclinical studies are required to assess the efficacy and pharmacokinetics of this intriguing formula.

## Data Availability

The datasets used and/or analyzed during the current study are available from the corresponding author on reasonable request.
